# Cell density and single-cell heterogeneity reveal distinct competence induction dynamics in the high-GC Gram-positive *Micrococcus luteus*

**DOI:** 10.1186/s12866-026-04757-7

**Published:** 2026-01-22

**Authors:** Antoni Lichev, Angel Angelov, Wolfgang Liebl

**Affiliations:** 1 Chair of Microbiology, Technical University of Munich, Emil-Ramann-Str. 4, 85354 Freising, Germany; 2 Present Address: Bioscience Core Laboratories, King Abdullah University of Science and Technology, Thuwal, 23955-6900 Kingdom of Saudi Arabia

**Keywords:** Natural transformation, Competence regulation, *Micrococcus luteus*, Single-cell analysis

## Abstract

**Background:**

Competence for natural transformation enables bacteria to acquire extracellular DNA and incorporate it into their genome, driving genetic diversification, DNA repair, and adaptation. While the regulatory mechanisms of competence development are well characterized in model organisms such as *Bacillus subtilis* and *Streptococcus pneumoniae*, little is known about how this process is controlled in *Actinomycetota*. Here, we investigate competence development in *Micrococcus luteus*, a high-GC Gram-positive species historically recognized for natural transformation.

**Results:**

Using transformation frequency assays, transcriptional reporters, single-cell flow cytometry, and fluorescence microscopy, we show that in this actinobacterial model competence is consistent with a probabilistic regulatory strategy that integrates cell density, nutrient-limitation-responsive signals, and physiological state. Peak transformation occurs during exponential growth in minimal medium at moderate inoculation densities, whereas both low and high starting densities suppress competence. Although transcription of the late competence genes *comEA/EC* is induced under competence-promoting conditions, this activation does not always correlate with transformability, indicating additional post-transcriptional or physiological regulation. Single-cell analyses revealed that promoter activity develops gradually and heterogeneously across the population, lacking the bistability or strong population-level coordination observed in other well-studied Gram-positive model systems.

**Conclusions:**

These data characterize competence induction dynamics in *M. luteus* and expand our understanding of the diversity of competence regulation across bacteria. While these observations constrain plausible regulatory models—supporting density- and nutrient-sensitive, probabilistic induction with heterogeneous single-cell activation—the upstream signal(s) or regulatory cascade controlling competence in *M. luteus* remain to be identified. Together, the results suggest that high-GC Gram-positive *Actinomycetota* may employ distinct, potentially bet-hedging-like strategies to balance growth, stress responses, and horizontal gene transfer.

**Supplementary Information:**

The online version contains supplementary material available at 10.1186/s12866-026-04757-7.

## Background

Natural competence is a physiological state that enables bacteria to undergo natural transformation by taking up extracellular DNA and incorporating it into the genome [1, 2]. This process promotes genome plasticity, DNA repair, and adaptation to changing environments [1, 2]. Despite the evolutionary divergence of competent species, the core DNA uptake machinery is highly conserved [1, 3]. It typically involves surface binding of extracellular DNA, transport across the cell membrane via ComEA and ComEC orthologs, and chromosomal integration mediated by RecA-like proteins [2, 4, 5].

While the molecular machinery involved in natural transformation is broadly conserved, the regulatory strategies that govern competence induction differ markedly across bacteria [4]. In *Bacillus subtilis*, a model organism of the phylum *Bacillota* (low-GC Gram-positive bacteria), stochastic activation of the master regulator ComK together with nutritional signals generates a bistable regime, in which only a subpopulation of cells enters competence [2, 6, 7]. In *Streptococcus pneumoniae*, also a *Bacillota* representative, competence is controlled by competence-stimulating peptide (CSP) signaling via the ComABCDE/ComDE circuit, producing a transient competence episode at the population scale [8–10]. While CSP signaling was historically interpreted as a quorum-sensing mechanism, competence output is strongly shaped by environmental conditions and cell history [8–10], and recent work indicates that competence can emerge from stochastically arising self-induced cells and then propagate through the population, promoting phenotypic heterogeneity rather than strict synchronization [11]. By contrast, several Gram-negative species belonging to the phylum *Pseudomonadota* — including *Vibrio cholerae*, *Haemophilus influenzae*, and *Helicobacter pylori* — do not rely on such strong positive-feedback loops. Instead, they exhibit environmentally triggered, often gradual and heterogeneous competence responses rather than tightly coordinated population-wide induction [12–14]. These examples highlight the diversity of bacterial strategies for aligning competence with environmental and population cues [1, 2].

In comparison, competence regulation in the *Actinomycetota* (also known as the high-GC Gram-positive bacteria) remains poorly understood [1]. *Micrococcus luteus*, a soil-dwelling, high-GC Gram-positive bacterium, was among the first species observed to undergo natural transformation [15, 16]. Its genome encodes homologs of key uptake proteins (ComEA and ComEC) as well as two *tad* (tight adherence) loci required for Flp pilus assembly, which are essential for transformation in this species [17]. However, *M. luteus* lacks several hallmark regulators of the best-characterized Gram-positive competence circuits (e.g., ComK, ComX in *Bacillus* and CSP-based signaling in *Streptococcus*), and does not encode type IV (pseudo)pili characteristic for the low-GC Gram-positive bacteria or the dynamic type IV pili typically linked to DNA uptake in Gram-negative bacteria [17]. Prior studies suggest that competence in *M. luteus* is promoted under nutrient limitation and during exponential growth [15–18], but how population-level and environmental signals are integrated remains unresolved.

In this study, we investigate the regulation of natural competence in *M. luteus* using a combination of transformation frequency assays, transcriptional reporters, flow cytometry, and fluorescence microscopy. To contextualize our findings relative to other naturally transformable model bacteria, we tested how competence induction depends on growth dynamics, inoculation density, and nutrient availability, and how *comEA/EC* transcriptional activation relates to transformability. We further quantified the distribution and dynamics of competence development across the population by single-cell analyses. Overall, the work characterizes a distinct competence induction strategy in the *Actinomycetota* representative *M. luteus* and expands our understanding of the diversity of natural transformation systems in bacteria.

## Methods

### Bacterial strains and growth conditions

All strains used in this study are listed in Table 1. Unless otherwise noted, strains were derived from *Micrococcus luteus* trpE16, a tryptophan auxotrophic strain of “*M. lysodeikticus*” ISU59 [19]. Cultures were grown at 30 °C with shaking (180 rpm) in either lysogeny broth (LB; Lennox formulation [20]) or glutamate minimal medium (MM; Wolin and Naylor [21]). LB contained 10 g/L peptone, 5 g/L yeast extract, and 5 g/L NaCl; MM contained 2 g/L K₂HPO₄, 1 g/L NH₄Cl, 10 g/L sodium glutamate, 7 g/L glucose, 0.1 g/L MgSO₄, 0.004 g/L FeSO₄, and 0.002 g/L MnCl₂. Media were adjusted to pH 7.2 with HCl and supplemented with 0.1 mg/mL tryptophan unless indicated. For solid cultivation, agar was added at 13 g/L. Where required, media were supplemented with 60 µg/mL kanamycin sulfate.

**Table 1:** List of bacterial strains used in this study

***M. luteus*** s**train**	**Synonyms**	**Genotype and relevant phenotype**	**Source**
ATCC 27141		“*Micrococcus lysodeikticus*” ISU; Trp^+^	Kloos, 1969a [15]
trpE16	wild-type	*trpE16*; mutagenesis derivative of ATCC 27141; Trp^−^	Kloos and Rose, 1970 [19]
trpE16 Δ01920*:eYFP*	Δ01920*:eYFP*	trpE16 Δ01920::*eYFP*-*kan*; Kan^R^; eYFP expression	This study
trpE16 Δ01920:*tdTomato*	Δ01920:*tdTomato*	trpE16 Δ01920::*tdTomato*-*kan*; Kan^R^; tdTomato expression	This study
trpE16 Δ*comEA/EC:eYFP*	Δ*comEA/EC:eYFP*	trpE16 Δ*comEA/EC*::*eYFP*-*kan*; Kan^R^; eYFP expression	This study
trpE16 Δ*comEA/EC*:*lacZ*	Δ*comEA/EC*:*lacZ*	trpE16 Δ*comEA/EC*::*lacZ-kan*; Kan^R^; LacZ expression	Lichev et al., 2019 [18]
trpE16 Δ*comEA/EC*:*tdTomato*	Δ*comEA/EC*:*tdTomato*	trpE16 Δ*comEA/EC*::*tdTomato*-*kan*; Kan^R^; tdTomato expression	This study

For transformation frequency assays, casein hydrolysate (CAH) agar was used, consisting of 1% (w/v) sodium glutamate, 0.2% (w/v) K₂HPO₄, 0.1% (w/v) NH₄Cl, 0.01% (w/v) MgSO₄, 0.0004% (w/v) FeSO₄, 0.0002% (w/v) MnCl₂, 0.5% (w/v) tryptophan-free acid hydrolyzed casein (EMD Millipore), 0.7% (w/v) glucose, and 1.3% (w/v) agar.

### Reporter strain construction

Reporter strains were constructed in *M. luteus* trpE16 by natural transformation and homologous recombination. Linear DNA fragments were assembled containing ~ 1 kb homology arms flanking the target locus, the reporter gene, and a Tn5 kanamycin resistance cassette [22]. The upstream homology arm ended 1 bp upstream of the target start codon, placing reporter expression under control of the native promoter. DNA fragments were PCR-amplified and assembled in vitro using a 20 µL Gibson Assembly reaction [23]. Competent *M. luteus* cells were directly transformed with the assembly mixture, and transformants were selected on LB agar containing 60 µg/mL kanamycin.

Using this strategy, multiple transcriptional reporter strains were generated. The promoter of the late competence operon (*comEA/EC*) was fused to three reporters: (i) *lacZ* from *E. coli*, amplified from vector pMKO [24]; (ii) *eYFP*, amplified from vector pEYFP; and (iii) *tdTomato*, amplified from plasmid pASTA3 (Addgene plasmid #24657 [25]). As a control, the promoter of Mlut_01920 (encoding an α-glucosidase predicted to show homogeneous expression) was fused to the ORF of either *eYFP* or *tdTomato*.

### Transformation frequency assays

Transformation was assessed by restoration of prototrophy in the tryptophan auxotroph *M. luteus* trpE16. For inoculation-density experiments, cells were grown in LB, harvested, washed, and inoculated into minimal medium (MM) at the indicated starting OD_600_ values. Transformability at 0 h was determined from the LB pre-culture prior to transfer into MM. For transformation assays, cells were harvested, washed, and resuspended in transformation buffer (100 mM CaCl₂, 50 mM Tris, pH 7.0). For each assay, 300 ng of plasmid pJET-*trpE*, carrying the wild-type *trpE* allele from *M. luteus* ATCC 27141, were added. Cultures were incubated for 30 min at 30 °C with shaking, chilled on ice, and sonicated (1 min, 30% amplitude, 0.25 duty cycle) using a UP200S ultrasound processor equipped with an S3 sonotrode (Hielscher Ultrasonics GmbH, Teltow, Germany) to disperse aggregates.

Aliquots were spread on CAH agar to select Trp⁺ transformants and on LB agar to determine total viable counts. Transformation frequencies were calculated as Trp⁺ CFU per total CFU. Negative controls without added DNA were included to determine the background level of spontaneous reversion, which defined the detection limit of the assay.

### Promoter activity assay

Promoter activity of the *comEA/EC* locus was assessed using the transcriptional reporter strain Δ*comEA/EC*:*lacZ*, as previously described and validated in detail by Lichev et al. [18]. In brief, washed precultures were transferred to 96-well microtiter plates and supplemented with the β-galactosidase substrate 4-methylumbelliferyl β-D-galactopyranoside (MUG; 100 µg/mL) to monitor LacZ activity, and Nile Red (20 µg/mL) as a growth-normalization dye.

Fluorescence was measured kinetically at 30 °C in a FLUOstar Omega microplate reader (BMG LABTECH, Germany), with MUG detected at 355/460 nm and Nile Red at 544/620 nm. MUG fluorescence was normalized to Nile Red fluorescence, and relative promoter activity was quantified by fitting four-parameter logistic functions to the normalized curves. The slopes of the fitted curves were used for comparison.

### Flow cytometry

Flow cytometry (FC) was performed using a BD LSRFortessa flow cytometer (BD Biosciences, USA). Cell suspensions with an OD₆₀₀ of 0.05–0.1 were prepared by pelleting cultures (13,000 × g, 5 min, 4 °C), washing with Milli-Q water (MQ), and resuspending in 1 mL MQ. To disrupt aggregates, samples were sonicated (1 min, 30% amplitude, 0.25 duty cycle) using a UP200S ultrasound processor with an S3 sonotrode (Hielscher Ultrasonics GmbH, Teltow, Germany). Data were acquired at 2000–20,000 events/s, with ≥ 100,000 events collected per sample. For the Δ*comEA/EC*:*tdTomato* and Δ01920:*tdTomato* reporter strains, fluorescence was measured using a 561 nm laser and a 586/15 PE-A filter. The parental strain *M. luteus* trpE16 served as a negative control.

### Fluorescence microscopy

Fluorescence microscopy was performed using an Axio Imager M1 epifluorescence microscope equipped with an AxioCam MRm monochrome digital camera and AxioVision software (Carl Zeiss AG, Germany). Prior to imaging, bacterial suspensions were sonicated (1 min, 30% amplitude, 0.25 duty cycle) to disperse aggregates. Because this dispersal disrupts native spatial relationships between cells, the microscopy assay was designed to quantify single-cell fluorescence distributions rather than to infer cell–cell proximity effects or spatial clustering. Cells were immobilized on 2% (w/v) agarose pads (5 × 5 mm), prepared by solidifying melted agarose between microscope slides. Unstained cells were visualized by differential interference contrast (DIC) microscopy. Fluorescence imaging was performed with the Δ*comEA/EC*:*eYFP* and Δ01920:*eYFP* reporter strains, while the parental strain *M. luteus* trpE16 served as a negative control. eYFP fluorescence was detected using Zeiss filter set 17 (excitation 485/20 nm, emission 515–560 nm).

### Fluorescence image analysis

Images were analyzed using a custom Python pipeline (Python v3.10). All images were processed without brightness or contrast adjustments to preserve raw signal integrity. To minimize edge artifacts, analysis was restricted to a circular region centered in each image (radius = 50% of image width/height). For segmentation, images were normalized using a 2nd–98th percentile contrast stretch and processed with the StarDist2D v0.9.1 pretrained model (2D_versatile_fluo) [26]. Segmented objects were filtered to exclude the smallest 3% by area, thereby removing debris and partial detections. Mean fluorescence intensity of each cell was extracted from the raw images and normalized on a per-image basis by min–max scaling to express relative intensity (0–100%). Normalized intensities were aggregated by strain and timepoint and visualized as beeswarm plots to display single-cell expression distributions.

## Results

### Competence induction depends on inoculation density and growth phase

**Fig. 1:**
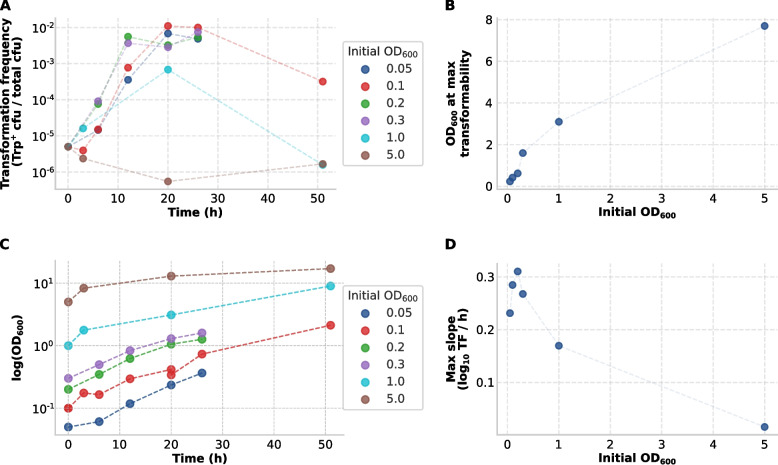
Dynamics of competence development in *M. luteus* trpE16 as a function of initial inoculation density. **A** Transformation frequency (Trp-prototrophic cfu/total cfu; log10 scale) is plotted over time for cultures inoculated at different initial OD_600_ values, ranging from 0.05 to 5.0. The 0 h value was measured in the LB pre-culture prior to transfer into MM. Cultures with moderate initial densities (OD_600_ = 0.1–0.3) exhibited faster and more pronounced increases in transformability compared to those started at low or very high densities. **B** Optical density (OD_600_) at the time point of maximal transformation frequency is shown for each initial inoculum. The wide range of OD_600_ values at which peak competence occurs indicates that transformability is not triggered at a fixed cell density threshold. **C** Growth curves showing log-transformed optical density (log_10_OD_600_) over time for the different cultures inoculated at different initial OD_600_ values. **D** Maximum competence induction rate (slope of log_10_-transformed transformation frequency per hour) plotted against initial OD_600_. The highest rates were observed at moderate inoculation densities, while only minimal competence induction occurred in cultures started at OD_600_ = 5.0

We first assessed how the initial inoculation density affects competence dynamics. *M. luteus* cultures inoculated into minimal medium (MM) at starting OD_600_ values ranging from 0.05 to 5.0 were monitored for transformability over time (Fig. 1A). The 0 h transformation frequency was measured in the LB pre-culture prior to washing and transfer to MM. Peak transformation frequencies (~ 10⁻^2^) were reached during mid-to-late exponential growth in cultures inoculated at moderate densities (OD_600_ = 0.2–0.3). Lower densities resulted in delayed competence induction, while cultures started at very high density (OD_600_ = 5.0) remained at or below the assay’s detection limit (≤ 10^⁻9^), defined by the spontaneous Trp⁺ reversion rate in negative controls, throughout the time course. By 20–26 h, most cultures (except OD_600_ = 5.0) converged to similar transformation frequencies (Fig. 1A), despite differences in their OD_600_ values.

Interestingly, the OD_600_ at which competence peaked varied substantially, ranging from ~ 0.2 to over 3.0 (Fig. 1B), indicating that competence was not triggered at a fixed cell density threshold. The corresponding growth curves for the different inoculation densities are shown in Fig. 1C. Because *M. luteus* trpE16 grows in MM with an estimated doubling time of ~ 8 h, cultures initiated at different OD_600_ values were sampled at different growth states at fixed time points, which should be taken into account when interpreting differences in transformability across inoculation densities. Together, these observations indicate that the timing and extent of competence induction depended on both the initial inoculation density and the subsequent growth dynamics. This argues against a simple single-threshold model based only on cell density (e.g., accumulation of a single secreted signal molecule) and suggests that density-dependent cues are integrated with the physiological state of the cells. Rather than being driven by a sharply defined signaling threshold, competence appears to emerge gradually in response to accumulating environmental or metabolic changes.

To further examine the kinetics of competence development, the maximum slopes of log-transformed transformation frequency were calculated as a proxy for induction rate (Fig. 1D). The steepest slopes, reflecting the most rapid competence onset, were observed in cultures inoculated at OD_600_ = 0.1–0.3, while slower induction occurred at OD_600_ = 0.05, and negligible induction was observed at OD_600_ = 5.

### Transcriptional activation of *comEA/EC* varies with inoculation density

**Fig. 2:**
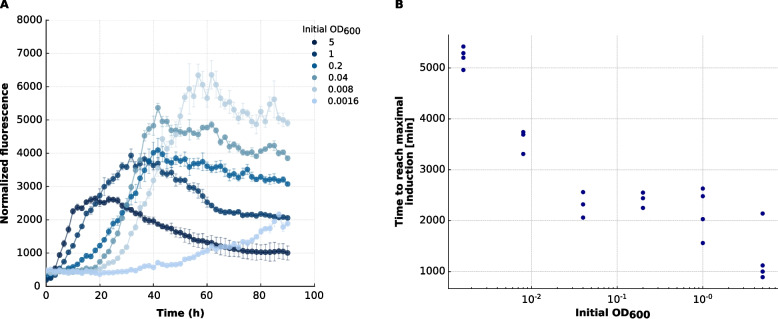
Density-dependent activation kinetics of the *comEA/EC* promoter in *M. luteus* trpE16 Δ*comEA/EC:lacZ* grown in MM. **A** Time courses of LacZ reporter activity for cultures inoculated at different initial OD₆₀₀ values (0.0016, 0.008, 0.04, 0.2, 1, 5). The reporter expresses the full-length *E. coli lacZ* ORF under the native *comEA/EC* promoter. LacZ activity—reported by MUG (4-methylumbelliferyl-β-D-galactopyranoside) fluorescence measured at 355/460 nm—was normalized to Nile Red fluorescence (544/620 nm) to account for differences in growth. Data points show the median across four biological replicates (*n* = 4); error bars indicate the interquartile range (IQR). Signal plateaus and subsequent decreases likely reflect MUG substrate depletion and limit further quantitative interpretation. **B** Time to reach maximum *comEA/EC* promoter induction (“peak”) as a function of initial inoculation density. “Peak” is defined as the time point at which the normalized fluorescence curve attains its steepest slope. Each data point represents a single biological replicate

To investigate the transcriptional regulation of late competence genes, we monitored expression of a *lacZ* reporter driven by the native *comEA/EC* promoter in cultures inoculated at varying starting OD_600_ values (0.0016–5.0) in MM. LacZ activity was measured over time using a MUG (4-methylumbelliferyl-β-D-galactopyranoside)-based fluorometric assay and normalized to Nile Red fluorescence to account for differences in growth (Fig. 2A). All cultures showed a steady increase in promoter activity, with higher initial cell densities leading to faster activation kinetics and steeper induction slopes.

Interestingly, even cultures inoculated at OD_600_ = 5.0—which failed to yield transformants—exhibited robust transcriptional activation of *comEA/EC*, indicating that transcription of these late competence genes is necessary but not sufficient for functional transformation. The time to reach peak promoter induction, defined as the steepest slope in the normalized fluorescence curve, was inversely correlated with inoculum size (Fig. 2B), consistent with density-dependent control but distinct from CSP-like positive-feedback circuits that can generate threshold-like, population-scale competence pulses via self-induction and propagation [11].

Control experiments showed that this promoter activation was specific to MM conditions and did not occur to the same extent in rich medium or under the control conditions (Additional File 1). While promoter activity was also detectable in rich medium, overall LacZ levels were much lower, suggesting that full activation of the *comEA/EC* promoter requires nutrient-limitation-responsive signals, consistent with previous reports [17, 18]. The observed uncoupling between transcriptional activation and transformation efficiency at high OD_600_ supports the presence of additional regulatory layers beyond transcription, potentially involving post-transcriptional control, stress responses, or inhibition of assembly of the DNA uptake machinery.

### Single-cell flow cytometry reveals gradual and heterogeneous competence induction

**Fig. 3:**
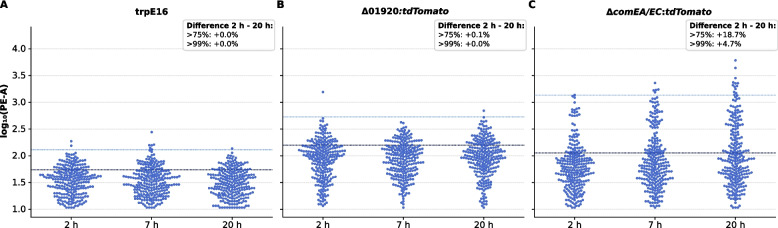
Flow cytometric analysis of PE-A fluorescence distribution shifts in *M. luteus comEA/EC* reporters over time. **A**–**C** Beeswarm plots showing single-cell distributions of log_10_-transformed PE-A fluorescence intensities for *M. luteus* trpE16 (**A**), Δ01920:*tdTomato* (**B**), and Δ*comEA/EC*:*tdTomato* (**C**) at 2 h, 7 h, and 20 h post-inoculation. For each strain and timepoint, 250 flow-cytometry events were randomly selected from the total measured population to provide equal sampling across conditions while preserving representative distributional features. Dashed horizontal lines indicate the 75th (dark blue) and 99th (light blue) percentiles of the 2 h distribution for each strain, used as internal thresholds to identify moderately and strongly fluorescent cells, respectively. Text boxes indicate the percentage-point increase (Δ 20 h – 2 h) in the fraction of cells exceeding these thresholds. These distributions show a gradual enrichment of highly fluorescent cells in the upper tail of the Δ*comEA/EC*:*tdTomato* population by 20 h, whereas the wild-type and Δ01920:*tdTomato* controls remain largely unchanged

Single-cell flow cytometry of strain *M. luteus* trpE16 Δ*comEA/EC:tdTomato*, a strain with a *tdTomato* reporter driven by the native *comEA/EC* promoter, revealed that competence induction in *M. luteus* is gradual and heterogeneous (Fig. 3). Cultures were sampled at 2 h, 7 h, and 20 h post-inoculation under competence-inducing conditions in MM. Fluorescence distributions broadened over time, with a growing upper tail of high-expressing cells specifically in the trpE16 Δ*comEA/EC:tdTomato* strain. In contrast, the control strains (trpE16 and trpE16 Δ01920*:tdTomato*, the latter carrying a reporter under the constitutively expressed Mlut_01920 promoter) maintained narrow, symmetrical distributions with low background fluorescence throughout the experiment.

Rather than a classic bimodal distribution, the Δ*comEA/EC:tdTomato* reporter strain exhibited a unimodal fluorescence profile that progressively skewed toward higher values. This suggests that competence gene activation does not occur via an abrupt switch but follows a probabilistic and asynchronous process across the population. The absence of a distinct subpopulation with sharply elevated expression may result from gradual induction kinetics, heterogeneous activation thresholds, or intrinsic biological variability in the reporter output.

To quantify changes over time, we calculated the fraction of cells exceeding defined fluorescence thresholds—specifically the 75th and 99th percentiles of the 2-h fluorescence distribution for each strain. After 20 h, approximately 4.7% of trpE16 Δ*comEA/EC:tdTomato* cells exceeded the 99th percentile threshold, and ~ 18% exceeded the 75th percentile, marking a substantial enrichment of high-expressing cells (Fig. 3). In contrast, the control strains showed negligible changes in these metrics.

Together, these findings confirm that *comEA/EC* promoter activity is induced in a fraction of cells in response to competence-inducing conditions. The observed distributional skew and time-dependent increase in high-fluorescence cells support a model of stochastic, density-sensitive regulation that does not match the bistable competence network of *B. subtilis* [27–29] or the CSP-mediated self-induction/propagation circuit described in *S. pneumoniae* [11].

### Fluorescence microscopy confirms heterogeneous single-cell induction of *comEA/EC*

To independently assess single-cell heterogeneity in competence gene expression, we performed fluorescence microscopy on a *M. luteus* reporter strain expressing *eYFP* under the native *comEA/EC* promoter (trpE16 Δ*comEA/EC:eYFP*). Two control strains were included: a wild-type trpE16 strain (non-fluorescent) and a constitutively fluorescent trpE16 Δ01920*:eYFP* strain, in which eYFP expression is driven by the promoter of the predicted alpha-glucosidase gene Mlut_01920. Cultures were grown in MM under competence-inducing conditions, and images were acquired at 0 h, 8 h, and 24 h post-inoculation.

**Fig. 4:**
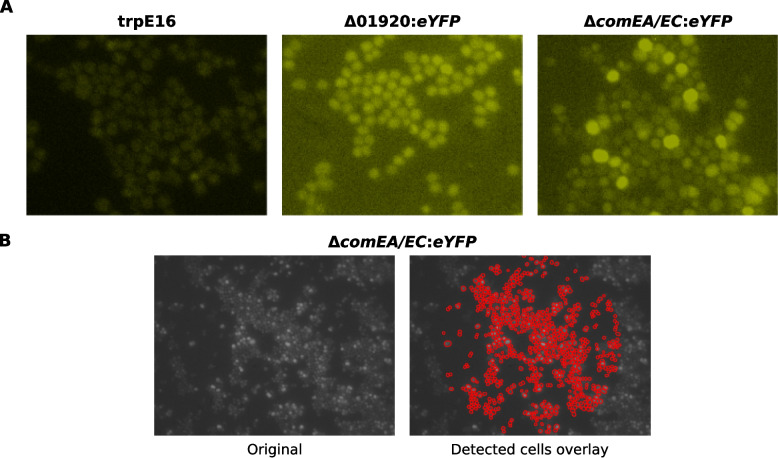
Representative fluorescence microscopy images and cell segmentation in *M. luteus* reporter strains. **A** Representative eYFP (excitation 485/20 nm, emission 515–560 nm) fluorescence images of the wild-type strain trpE16, the constitutive reporter strain trpE16 Δ01920:*eYFP*, and the competence-related reporter strain trpE16 Δ*comEA/EC*:*eYFP* under competence-inducing conditions (growth in MM). The grayscale fluorescence images were pseudo-coloured to enhance visual contrast and make differences in fluorescence intensity more apparent, making heterogeneity in *comEA/EC* promoter activity within and between strains more apparent. Images were acquired with identical microscope acquisition settings; subsequent brightness and contrast adjustments were performed for visualization only. **B** Fluorescence microscopy image of the competence-related reporter strain trpE16 Δ*comEA/EC*:*eYFP* (left) and the corresponding segmentation overlay (right), in which detected single cells are highlighted in red. Cell detection was performed using the StarDist segmentation framework after contrast normalization to the 2nd–98th percentile, followed by size filtering to exclude the smallest 3% of objects. Only cells located within a central circular region of interest were included in the analysis to minimize edge artifacts and background variation. Together, these panels illustrate the typical distribution of eYFP signal and highlight the heterogeneous induction of the *comEA/EC* promoter under competence-inducing conditions

**Fig. 5:**
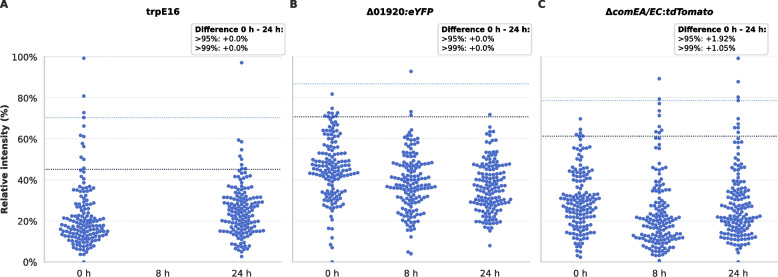
Single-cell fluorescence distributions of *M. luteus comEA/EC* reporter strains under competence-inducing conditions. **A**–**C** Beeswarm plots show single-cell relative fluorescence intensities for the wild-type strain trpE16, the constitutive expression control Δ01920:*eYFP*, and the competence reporter Δ*comEA/EC*:*eYFP* at 0 h, 8 h, and 24 h post-inoculation in MM. Each beeswarm displays 150 randomly selected cells per strain and timepoint to ensure comparable visual density across conditions. The underlying n-values, representing the total number of segmented cells, were: trpE16 (0 h: 3505; 8 h: 0; 24 h: 2892), Δ01920:*eYFP* (0 h: 4140; 8 h: 7684; 24 h: 2326), and Δ*comEA/EC*:*eYFP* (0 h: 9827; 8 h: 2675; 24 h: 2122). Dashed lines mark the 95th and 99th percentiles of the 0-h distribution for each strain. Text boxes report the percentage-point changes in the fraction of cells exceeding these thresholds (Δ 24 h – 0 h), calculated from all measured cells. The Δ*comEA/EC*:*eYFP* strain shows a clear broadening and upward skew of the fluorescence distribution over time, consistent with a heterogeneous activation of the *comEA/EC* promoter during competence development, whereas trpE16 and Δ01920:*eYFP* remain stable

Image segmentation and analysis were conducted using a StarDist-based [26] pipeline with per-image normalization (Fig. 4). Beeswarm plots of relative single-cell fluorescence intensities (Fig. 5) revealed that trpE16 and trpE16 Δ01920*:eYFP* displayed stable fluorescence distributions over time, as expected for negative and constitutive controls. In contrast, the trpE16 Δ*comEA/EC:eYFP* strain exhibited a progressive broadening of the fluorescence distribution, with an increasingly pronounced upper tail by 24 h, indicating activation of the *comEA/EC* promoter in a fraction of cells.

The fluorescence distributions remained unimodal but skewed, without clear bimodal separation. To quantify the induction, we defined expression thresholds based on the 99th and 95th percentiles of the 0 h distribution. At 24 h, 1.05% and 1.92% of trpE16 *ΔcomEA/EC:eYFP* cells exceeded these thresholds, respectively, while control strains showed negligible increases. These stringent percentile-based cutoffs were chosen to highlight the emergence of a high-expression tail and should not be interpreted as the total fraction of competent (transformable) cells. Notably, the presence of high-expressing cells at 0 h—corresponding to late stationary phase of the rich medium precultures—suggests low-level background activation of *comEA/EC* in a small fraction even before transfer to competence-inducing conditions.

These microscopy results closely mirrored flow cytometry findings and further support a model in which competence gene induction in *M. luteus* is gradual, heterogeneous, and governed by a density-dependent regulatory mode distinct from the best-characterized competence systems described in Gram-positive model bacteria (e.g., *B. subtilis* and *S. pneumoniae*). The absence of a distinct bistable switch and the heterogeneity in *comEA* expression are consistent with probabilistic competence activation across the population.

## Discussion

Our findings suggest that competence development in the actinobacterium *M. luteus* follows an integrative, probabilistic regulatory strategy. Peak transformation frequencies occurred during exponential growth in MM at moderate inoculation densities, while both very low and very high starting densities impaired or retarded competence (Fig. 1). The absence of a fixed cell density threshold argues against a cell-density-only trigger and instead supports integration of population cues with physiological state. The inoculation-density–dependent kinetics we observe (rapid onset at moderate density, delayed onset at low density, and no detectable induction at very high starting density; Fig. 1D) are qualitatively consistent with recent *S. pneumoniae* models in which competence initiates in a minority of self-induced cells and population-level output depends on density and local context [11]. However, the upstream logic in *M. luteus* is likely distinct, given the lack of canonical CSP–ComABCDE circuitry and the absence of evidence for rapid, population-wide competence propagation under our conditions.

Robust transcriptional activation of *comEA/EC* at high cell densities did not result in transformability, indicating regulatory layers beyond *comEA/EC* transcription. Possible mechanisms include post-transcriptional control under nutrient limitation or stress (e.g., translational repression or metabolic inhibition) and impaired assembly or function of the pilus–DNA translocase, any of which could allow *comEA/EC* promoter activation without commitment to competence, potentially acting as a safeguard under suboptimal conditions. Similar decoupling has been observed in *B. subtilis*, where (p)ppGpp signaling and alternative sigma factors modulate competence in response to stress [30–32]. In *M. luteus*, deleting both the predicted RelA-type protein and a small alarmone synthetase reduced transformability by ~ 2 orders of magnitude in MM, implicating (p)ppGpp-dependent control of competence [18]. Chemical induction of the stringent response with mupirocin or serine-hydroxamate repressed *comEA/EC* promoter activity, providing further evidence for the involvement of the stringent response [18]. Consistently, amino acid supplementation repressed both *comEA/EC* promoter activity and transformability, whereas amino acid scarcity in MM favored both [18]. Notably, *M. luteus* also encodes core DNA repair components (e.g., RecA and putative LexA/Uvr-family proteins), consistent with an SOS-like DNA damage response; in other bacteria, (p)ppGpp can potentiate RecA/LexA-controlled SOS induction, providing a plausible link between stringent response signaling, genome maintenance, and competence regulation. Together, these observations suggest that competence is tuned by global stress and nutrient-sensing pathways, consistent with the transcriptional and functional differences we observe across different inoculation densities.

Single-cell analyses by flow cytometry and fluorescence microscopy show that *comEA/EC* promoter activation in *M. luteus* increases gradually and remains heterogeneous across the population (Figs. 3, 4 and 5). In both assays, the distributions broaden over time and remain largely unimodal while becoming increasingly right-skewed, consistent with enrichment of a high-expression tail rather than an abrupt transition into a clearly separated competent versus non-competent state. This behavior contrasts with ComK-driven bistability in *B. subtilis*, where a strong positive-feedback loop can produce a discrete competent state in a subset of cells, and with pneumococcal competence dynamics controlled by CSP-dependent circuitry [8, 27–29]. Notably, the earlier “timing device” interpretation of pneumococcal CSP-dependent competence initiation has been challenged by evidence that competence onset depends strongly on cell density, environmental conditions, and cellular history [10]. More recent work further supports a biphasic self-induction and propagation (SI&P) framework in which competence stochastically initiates in a minority of self-induced “initiator” cells and is then amplified via CSP-dependent positive feedback, producing a transient population-scale competence episode that fosters phenotypic heterogeneity rather than strict synchronization [11]. In contrast, under our conditions we observe no clear bimodal separation in *M. luteus*, consistent with weak or absent switch-like positive feedback.

In this context, the presence of a small high-expression tail already at 0 h (late stationary phase of the LB pre-culture) is consistent with a general principle shared across competence models: rare cells can occupy an “initiator-like” or pre-activated state that precedes broader competence-associated transcription. In *B. subtilis*, such rare pre-competent states can arise from noise-driven fluctuations in basal *comK* expression and may be reinforced by ComK-dependent positive feedback in cells that cross a threshold [27, 33, 34]. In *S. pneumoniae*, the SI&P model likewise emphasizes a minority of self-induced initiator cells as the entry point for competence amplification and transmission through the population [11]. Moreover, because microscopy samples were sonicated prior to imaging to disperse aggregates for single-cell quantification, we cannot assess whether high-expressing cells were spatially clustered in the original culture or whether competence-related transcription preferentially arises among neighboring cells. Microfluidic time-lapse microscopy that preserves spatial neighborhoods under controlled medium exchange would be well suited to test proximity- or contact-dependent competence activation in *M. luteus*.

More broadly, competence regulation among *Bacillota* is more diverse than these two frequently cited models suggest. In ComRS-containing streptococci, competence can likewise be transient and strongly shaped by physiological context: in *S. thermophilus*, competence depends on a processed peptide signal that is secreted and re-imported and is sensitive to nutritional peptide availability [35], whereas in *S. salivarius*, competence shut-off can be implemented through intracellular degradation of the activating peptide signal, providing a built-in negative feedback that limits competence duration [36]. These ComRS-based systems therefore share with *M. luteus* the general principle that competence output can be strongly context-dependent and temporally restricted, rather than triggered at a single population-wide density threshold. Consistent with strong environmental gating, single-cell work in *S. mutans* also shows that the population-level output of *comX* can switch between unimodal and bimodal responses depending on growth medium [37]. A similarly physiology-centric logic has also been reported for *Lactococcus lactis*, where competence depends on activation of ComX but is controlled by global nutritional/stress regulators and proteolytic turnover via the MecA–Clp machinery, rather than by a dedicated peptide-based cell–cell communication system [38, 39]. Finally, in *Staphylococcus aureus*, competence has been described as condition-dependent and restricted to a minor subpopulation, with SigH-dependent activation enabling transformation and biofilm-associated signaling enhancing competence gene expression and transformation efficiency [40, 41].

In line with this broader diversity, our *M. luteus* data point to a gradual, probabilistic increase in competence across the population without evidence for relay-based propagation or abrupt transitions under our conditions. Notably, in contrast to many well-characterized model systems, we currently have no evidence for the involvement of a dedicated competence-inducing signaling peptide or a two-component system in *M. luteus*. Instead, competence activation increases gradually over time, with a growing fraction of cells inducing *comEA/EC* without an abrupt shift into a clearly separated state. This suggests a distinct form of population-level propagation governed by environmental signal accumulation (e.g., nutrient depletion or secreted metabolites) rather than secreted peptide–mediated positive feedback circuits that coordinate competence at the population level. The probabilistic regulation observed in *M. luteus* is consistent with an adaptive bet-hedging strategy, allowing fractions of cells to transiently enter the competent state while others continue growth [11, 42]. Such strategies may be evolutionarily advantageous in fluctuating environments where the benefits of transformation are unpredictable or short-lived. Because competence regulons in other bacteria can mediate functions beyond transformation and the competence regulon of *M. luteus* is currently unknown, competence may provide additional benefits that remain to be elucidated.

Overall, our results delineate population-level and single-cell features of competence induction in *M. luteus* and underscore that these dynamics differ from the varied models described in other organisms, while also highlighting that the upstream inducing cues and regulatory components remain to be identified. In particular, the identity of the signals that initiate competence development under MM conditions, the factors that sense and integrate these cues, and the step(s) that limit transformability at very high inoculation density despite strong *comEA/EC* transcription are not yet resolved. Addressing these unknowns will require targeted perturbations of candidate regulators and direct measurements of signal accumulation and competence apparatus assembly, guided by the constraints provided by our phenotypic and single-cell data.

## Conclusions

In conclusion, we show that competence development in *Micrococcus luteus* is governed by an integrative, probabilistic regime that depends on both inoculation density and growth dynamics rather than a fixed cell density threshold. Transcriptional activation of *comEA/EC* is necessary but not sufficient for transformability, and single-cell analyses reveal gradual, heterogeneous promoter induction without clear bistable or coordinated switching under the tested conditions. These observations constrain plausible regulatory models and establish *M. luteus* as a useful system for studying competence induction in high-GC Gram-positive *Actinomycetota*. However, the upstream signal(s), regulatory nodes, and post-transcriptional steps that couple promoter activation to assembly of a functional DNA-uptake apparatus remain to be identified.

## Supplementary Information

Additional file 1. Transcriptional activation of the *M. luteus* *comEA/EC* promoter across initial inoculation densities via LacZ reporter. Transcriptional activation of the *M. luteus* *comEA/EC* promoter across initial inoculation densities via LacZ reporter. Transcriptional activation of the *comEA/EC* promoter in *M. luteus* was monitored using a LacZ reporter strain (trpE16 Δ*comEA/EC:lacZ*) grown in MM at six different initial OD_600_ values (0.0016, 0.008, 0.04, 0.2, 1, 5). LacZ activity, detected via fluorescence resulting from MUG hydrolysis (measured at 355/460 nm), was normalized to Nile Red fluorescence (544/620 nm) to account for variations in cell density. Blue curves represent the median normalized fluorescence of 4 biological replicates per condition, with error bars indicating the interquartile range. Each panel displays a distinct control group for comparison: (A) trpE16 Δ*comEA/EC:lacZ* reporter strain grown in LB at the same six OD_600_ values, shown as red/orange curves (darker shading represents higher initial OD_600_ values; 4 replicates each). (B) trpE16 wild-type strain (lacking LacZ) grown in MM or LB at the same six OD_600_ values, shown as green dashed curves (single replicate per condition). (C) No-cell controls (medium only), shown as brown dashed curves. These include MM and LB with MUG, Nile Red, both dyes, or no dye added (3 replicates each). (D) trpE16 Δ*comEA/EC:lacZ* reporter strain and trpE16 wild-type strains grown in MM or LB at OD_600_ = 5 in the absence of dyes, shown as orange dashed curves (3 replicates each). Together, these controls confirm that the observed reporter signal results from active LacZ expression and demonstrate that background fluorescence from the medium or other sources is negligible.

## Data Availability

The datasets used and/or analyzed during the current study are available from the corresponding author on reasonable request.
