# Supplementary figures and images for: Cell density and single-cell heterogeneity reveal distinct competence induction dynamics in the high-GC Gram-positive Micrococcus luteus
Source: BMC Microbiol. 2026 Jan 22;26:63. doi: 10.1186/s12866-026-04757-7 (PMC12849477; doi:10.1186/s12866-026-04757-7)

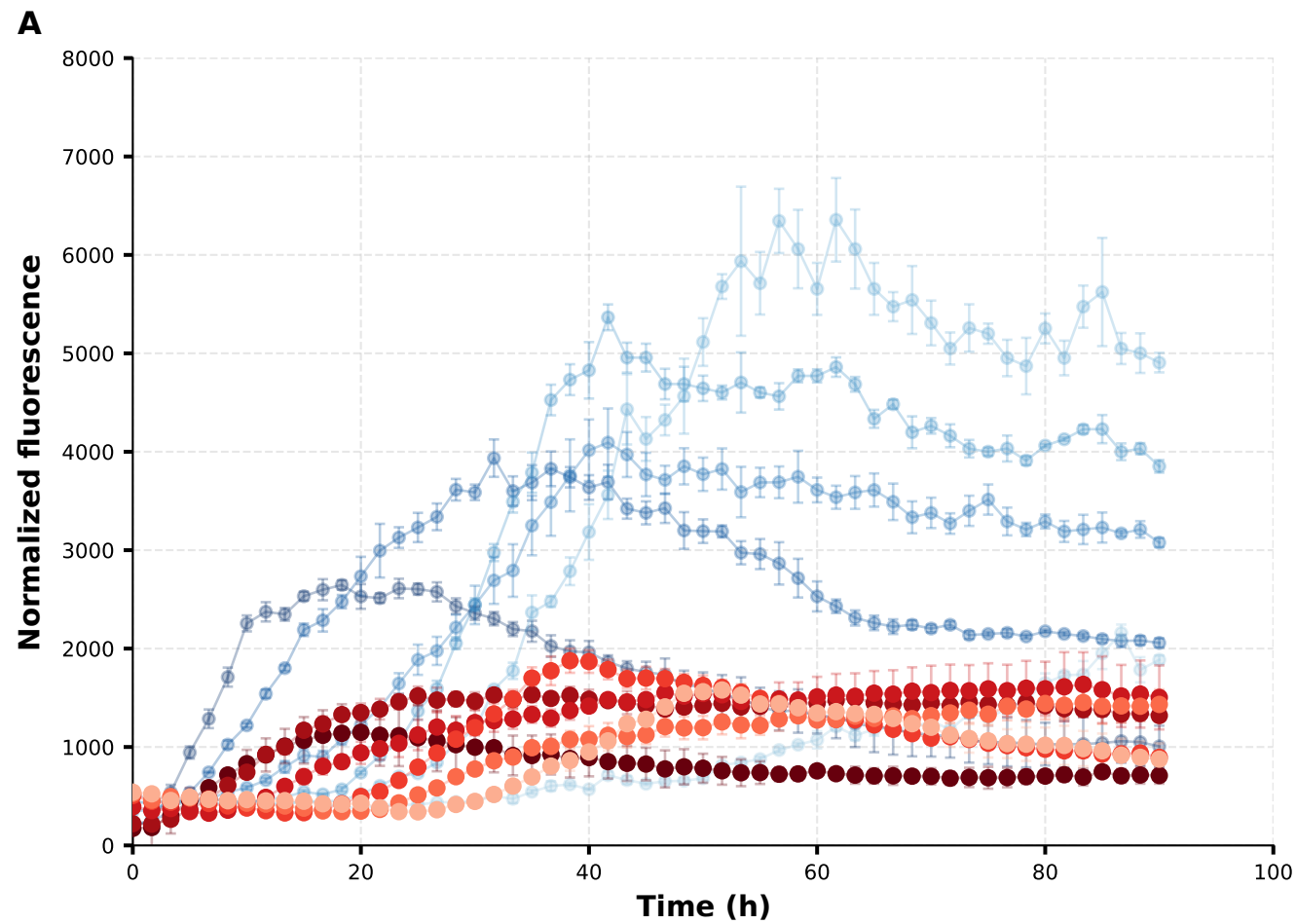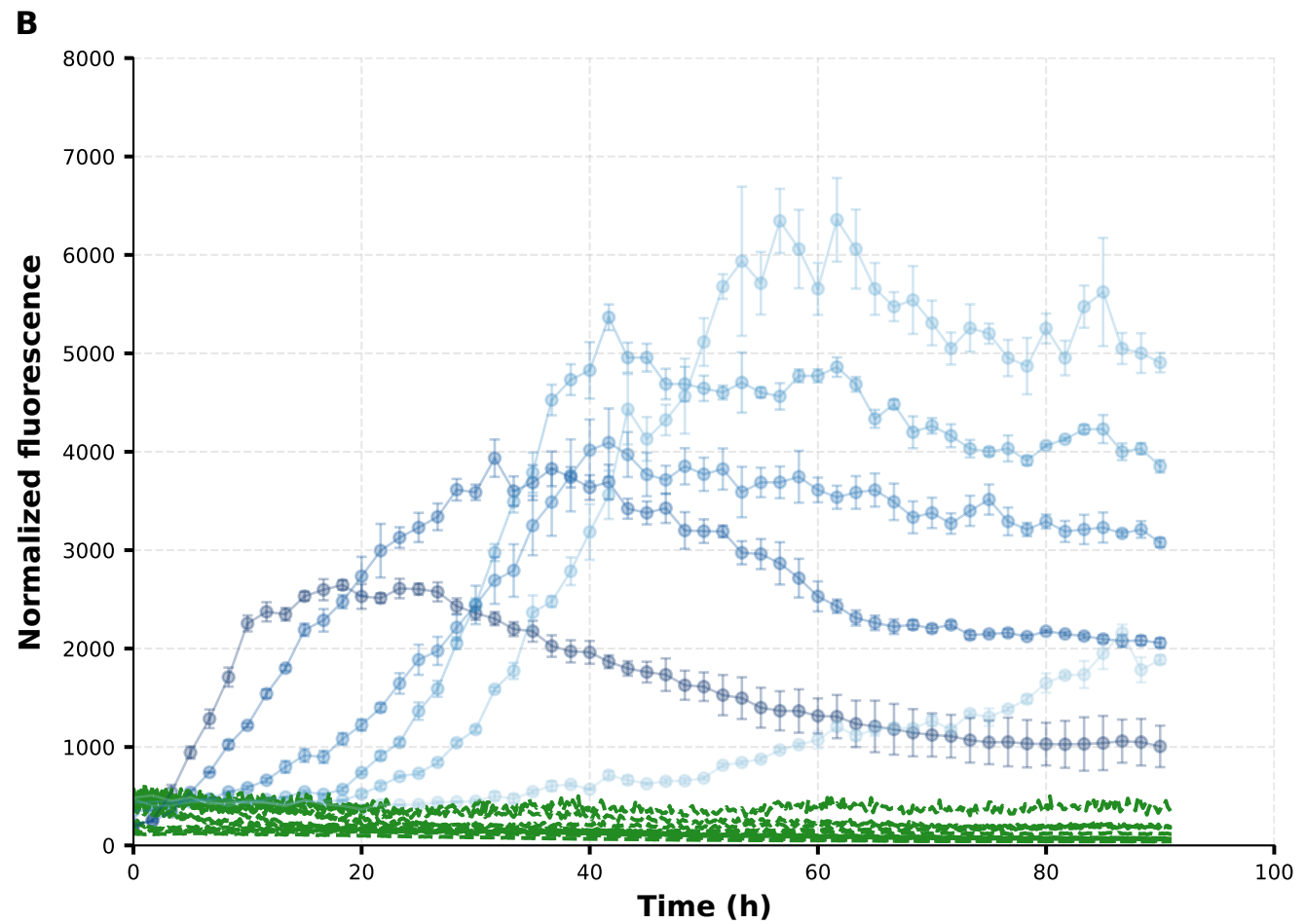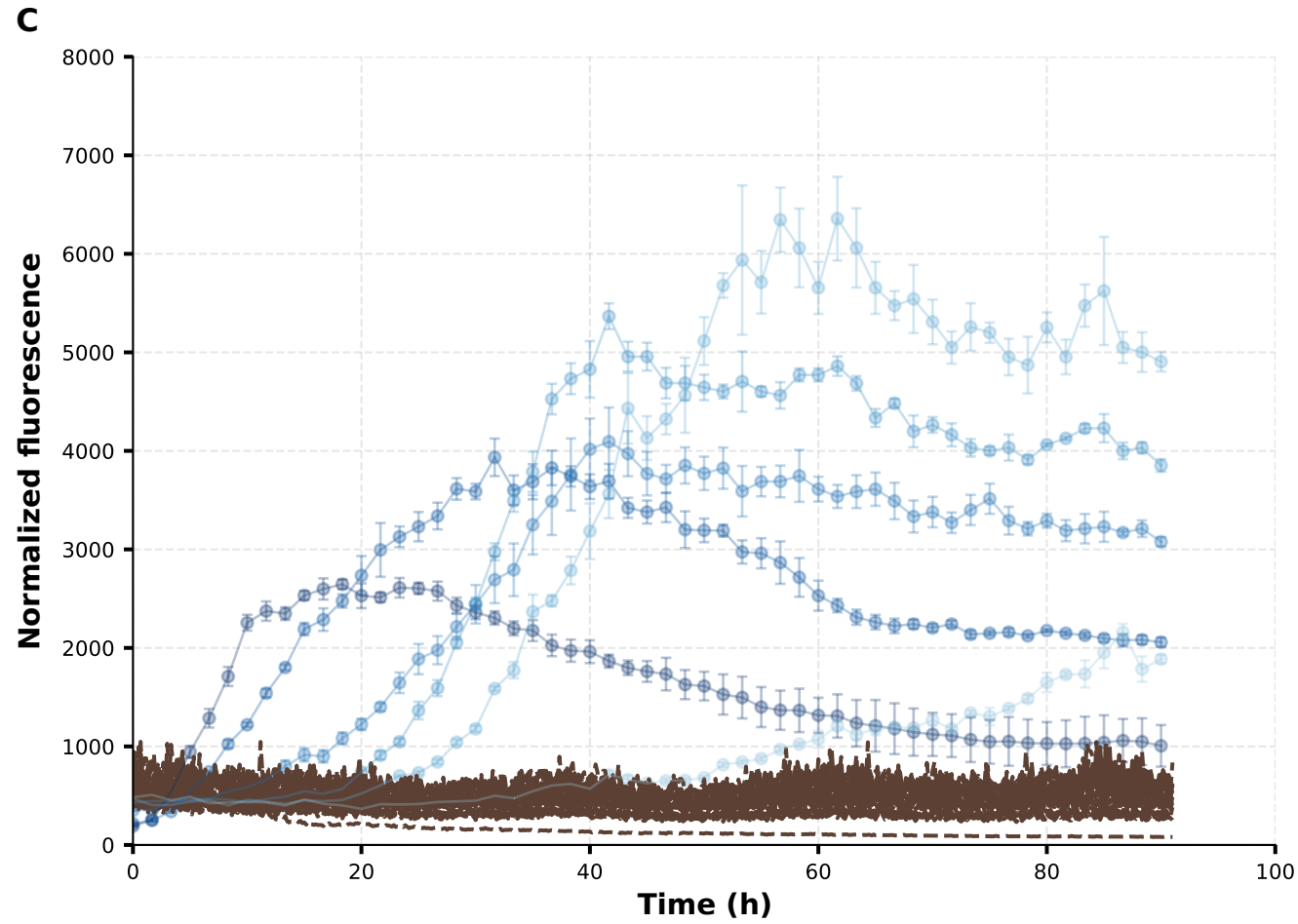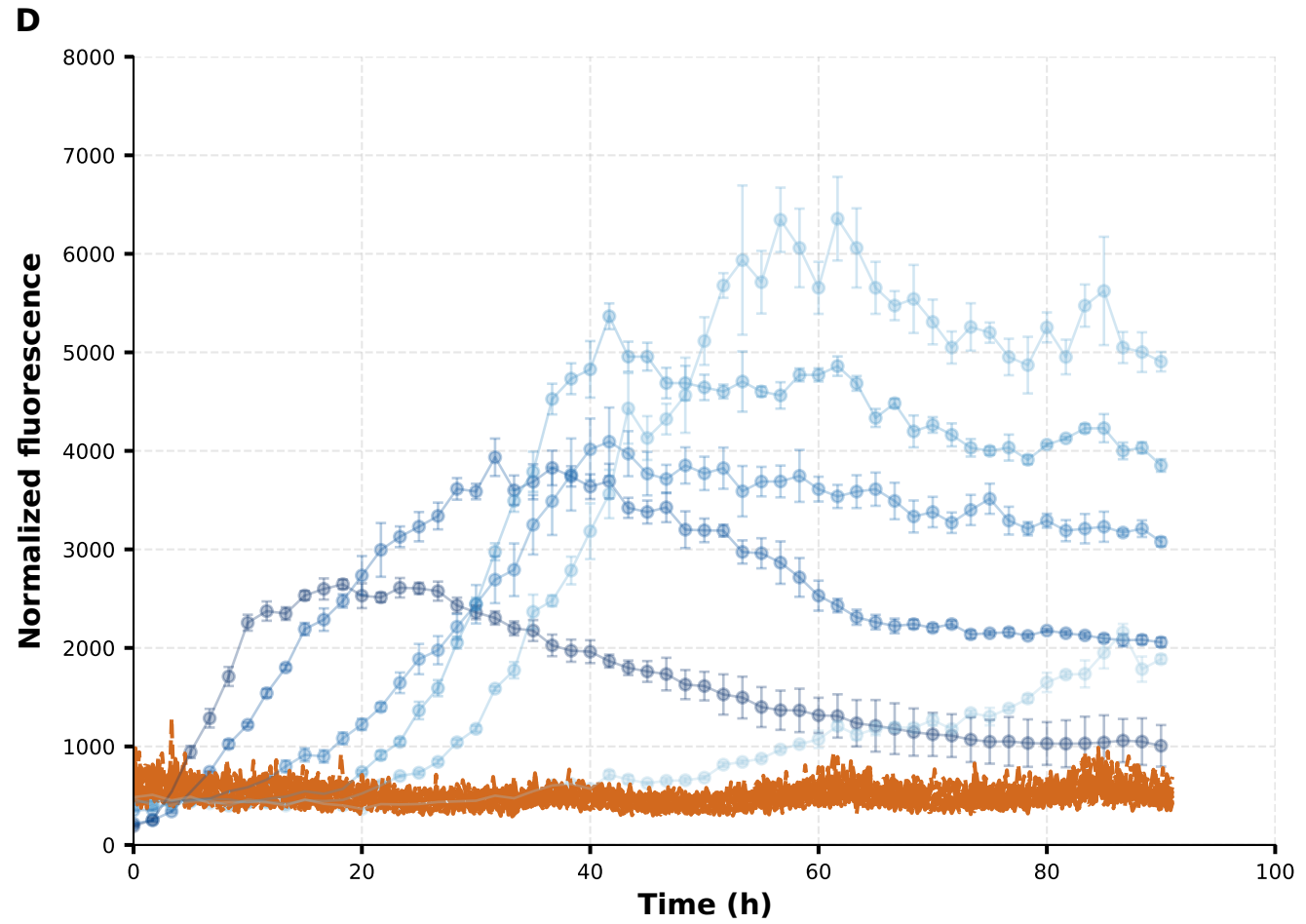

Supplement: Supplementary file 1 — Additional file 1. Transcriptional activation of the M. luteus comEA/EC promoter across initial inoculation densities via LacZ reporter. Transcriptional activation of the M. luteus comEA/EC promoter across initial inoculation densities via LacZ reporter. Transcriptional activation of the comEA/EC promoter in M. luteus was monitored using a LacZ reporter strain (trpE16 ΔcomEA/EC:lacZ) grown in MM at six different initial OD600 values (0.0016, 0.008, 0.04, 0.2, 1, 5). LacZ activity, detected via fluorescence resulting from MUG hydrolysis (measured at 355/460 nm), was normalized to Nile Red fluorescence (544/620 nm) to account for variations in cell density. Blue curves represent the median normalized fluorescence of 4 biological replicates per condition, with error bars indicating the interquartile range. Each panel displays a distinct control group for comparison: (A) trpE16 ΔcomEA/EC:lacZ reporter strain grown in LB at the same six OD600 values, shown as red/orange curves (darker shading represents higher initial OD600 values; 4 replicates each). (B) trpE16 wild-type strain (lacking LacZ) grown in MM or LB at the same six OD600 values, shown as green dashed curves (single replicate per condition). (C) No-cell controls (medium only), shown as brown dashed curves. These include MM and LB with MUG, Nile Red, both dyes, or no dye added (3 replicates each). (D) trpE16 ΔcomEA/EC:lacZ reporter strain and trpE16 wild-type strains grown in MM or LB at OD600 = 5 in the absence of dyes, shown as orange dashed curves (3 replicates each). Together, these controls confirm that the observed reporter signal results from active LacZ expression and demonstrate that background fluorescence from the medium or other sources is negligible. [file 12866_2026_4757_MOESM1_ESM.pdf]
